# Graphitized Carbon: A Promising Stable Cathode Catalyst Support Material for Long Term PEMFC Applications

**DOI:** 10.3390/ma11060907

**Published:** 2018-05-28

**Authors:** Paritosh Kumar Mohanta, Fabian Regnet, Ludwig Jörissen

**Affiliations:** Zentrum für Sonnenenergie-und Wasserstoff-Forschung Baden-Württemberg, Helmholtzstrsse 8, 89081 Ulm, Germany; fabian.regnet@zsw-bw.de (F.R.); ludwig.joerissen@zsw-bw.de (L.J.)

**Keywords:** PEMFC, MEA, catalyst support, catalysts, Pt, graphitized carbon

## Abstract

Stability of cathode catalyst support material is one of the big challenges of polymer electrolyte membrane fuel cells (PEMFC) for long term applications. Traditional carbon black (CB) supports are not stable enough to prevent oxidation to CO_2_ under fuel cell operating conditions. The feasibility of a graphitized carbon (GC) as a cathode catalyst support for low temperature PEMFC is investigated herein. GC and CB supported Pt electrocatalysts were prepared via an already developed polyol process. The physical characterization of the prepared catalysts was performed using transmission electron microscope (TEM), X-ray Powder Diffraction (XRD) and inductively coupled plasma optical emission spectrometry (ICP-OES) analysis, and their electrochemical characterizations were conducted via cyclic voltammetry(CV), rotating disk electrode (RDE) and potential cycling, and eventually, the catalysts were processed using membrane electrode assemblies (MEA) for single cell performance tests. Electrochemical impedance spectroscopy (EIS) and scanning electrochemical microscopy (SEM) have been used as MEA diagonostic tools. GC showed superior stability over CB in acid electrolyte under potential conditions. Single cell MEA performance of the GC-supported catalyst is comparable with the CB-supported catalyst. A correlation of MEA performance of the supported catalysts of different Brunauer–Emmett–Teller (BET) surface areas with the ionomer content was also established. GC was identified as a promising candidate for catalyst support in terms of both of the stability and the performance of fuel cell.

## 1. Introduction

Polymer electrolyte membrane fuel cells (PEMFC) are considered as a viable option for future zero emission mobility, due to their high power density and low degradation. However, long term operation of PEMFC is hindered due to instability of the traditional carbon black type catalyst support materials under fuel cell operating conditions. A material could be used as a noble metal supports if it meets some general requirements, for example, high surface area, high electronic conductivity, stable under acidic environment, uniform dispersion of noble metal nanoparticles on it, effective metal-support interaction, and low cost [1]. Currently, carbon black (CB) is widely used. However, the stability of CB is not high enough for long term fuel cell operation. In particular, during start and stop cycling of the fuel cell, CB in the cathode is rigorously oxidized, which causes detachment of Pt nanoparticles and promote agglomerations, and eventually, performance degradation [2,3,4]. Modified carbon, inorganic oxides, and composite materials are currently research areas of interest. For example, Selvaganesh et al. showed that using graphitized carbon as a support for Pt and its alloy was more stable than non-graphitized carbon [5]. Takei et al. [6] also proposed graphitized carbon as a cathode catalyst support. Modified graphitized carbon [7], or nitrogen and fluorine co-doped graphite nanofibers [8], are also promising support materials. Silica coated carbon nanotube [9], carbon nanofiber [10,11], composite of activated carbons [12], composite of xerogel-nanofiber carbon composites [13], nitrogen-containing carbon support [14], and graphitic hollow carbon nanocages [15] were just recently reported as stable cathode catalyst supports for PEMFC applications.

Besides CB, inorganic oxides, for example, TiO_2_, Magnéli-phase titanium oxide, and SnO_2_, are considered as stable cathode catalyst supports for PEMFC [16,17,18,19] although their electrical conductivities are too low. Metal oxides with appropriate doping can improve the electrical conductivities to a desired level. However, the main problem of doped metal oxides is their low Brunauer–Emmett–Teller (BET) surface areas and their low electronic conductivities compared to the CB support. Nevertheless, Sb-doped SnO_2_ [20,21,22,23], Nb-doped SnO_2_ [24], Nb-doped TiO_2_ [25,26], and In-doped TiO_2_ [27] are suggested as catalyst supports for PEMFC application. Catalyst supported on carbon-doped TiO_2_ also showed improved stability compared to catalysts supported on CB reported by Huang et al. [28] and Liu et al. [29]. Titanium diboride [30], titanium carbide [31], and very recently, tungsten carbide [32], are proposed as stable cathode catalyst supports for PEMFC. Unfortunately, most of the research work is limited to studies in liquid acid electrolyte, where the proposed supported catalysts are stable. Yet, to get a full picture, one should study the effects on cell performance as well. In our previous work, we showed the feasibility of using Sb doped SnO_2_ (ATO) as a cathode catalyst support, in which the catalyst supported on ATO was stable in acid electrolyte, but ultimately, the MEA single cell performance was low compared to a CB-supported material [33]. Thus, the aim of this work is to investigate alternative carbon-based supports that are more stable, without compromising fuel cell performances. A graphitized carbon and a traditional CB support were chosen for this investigation. Supported catalysts were prepared via an already developed modified polyol process. The prepared supported catalysts were annealed to increase their stability and activities [34,35], and then characterized. For comparison, a commercial high BET surface area (800 m^2^/g) CB-supported Pt electrocatalyst from Tanaka (TEC10E20E, Lot:1015-0041, Tanaka Kikinzoku Kogyo K.K. Tokyo, Japan, 19.3 wt % Pt,) was also taken as reference catalyst in this work. 

## 2. Results

### 2.1. Catalysts Characterization

Pt-loaded catalysts (20 wt %) were prepared on CB and GC supports via an already developed modified polyol process [33]. BET surface areas of the investigated CB and the GC supports were measured as 192 and 60 m^2^/g, and the electrical conductivities of the supports were measured as 2.7 and 2.1 S/cm, respectively. 

#### 2.2.1. Physical Characterization of the Synthesized Catalysts

**Figure 1:**
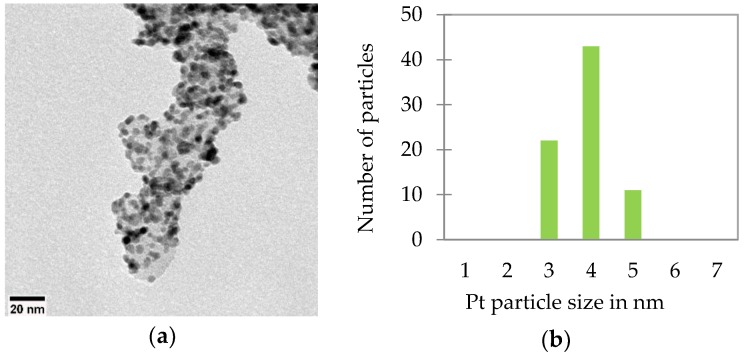
TEM image of a non-annealed 18.5 wt % Pt on CB supported synthesized catalyst (**a**) and distribution of Pt nanoparticle on the support (**b**).

Figure 1 shows a TEM image of a CB-supported synthesized catalyst (non-annealed). It reveals the homogeneous distributions of the Pt particles between 3 and 5 nm (average 3.9 nm). The average Pt particle size of the same catalyst was determined to be 3.2 nm via XRD, a good compromise of the measurement technique. Therefore, the average particle sizes of Pt for all of our synthesized catalysts were measured by the same way via XRD using TOPAS software (version 5, Bruker AXS, Karlsruhe, Germany). 

**Figure 2:**
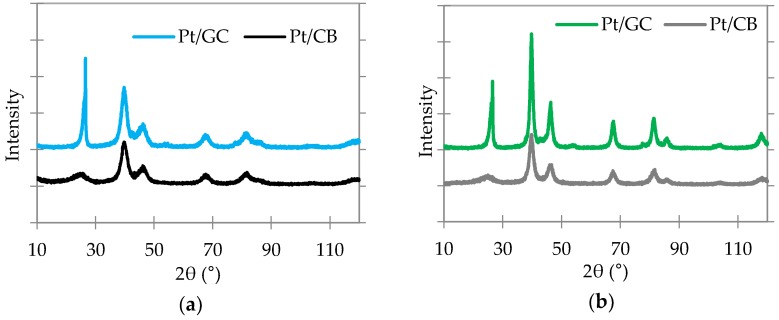
XRD patterns of the catalysts (**a**) before annealing and (**b**) after annealing.

Figure 2 shows the XRD patterns of the CB- and GC-supported catalysts before and after the annealing process, in which the first peak at 2θ value of 25° belongs to carbon (or graphite) and other reflexes at 39°, 46°, 68°, 81°, and 85°, correspond to Pt(111), Pt(200), Pt(220), Pt(311), and Pt(222) planes, respectively. This is a characteristic of cubic Pt crystallites [29]. After annealing, the Pt peaks intensities are increased, while the half width at half maximum decreased, which is an indication of increasing Pt nanoparticle sizes compared to non-annealed catalysts. The average Pt particle size of the CB- and the GC-supported non-annealed catalysts were measured by XRD as 3.0 and 2.9 nm, respectively, and the annealed catalysts were measured as 5.5 and 5.8 nm, respectively. Pt nanoparticle are located closer to each other on the supports with low BET surface areas (GC). Therefore, they have high tendency to become mobile and agglomerate. Thus, the increase of Pt particle size on GC support is higher than the CB support during annealing process. The average Pt particle size of the reference Tanaka catalyst was below the detectable limit (<2 nm) via XRD.

The total Pt content were respectively measured as 18.8 and 19.7 wt % from the CB- and the GC-supported synthesized catalysts after annealing via ICP-OES analysis. 

#### 2.2.2. Carbon Corrosion in Acid Electrolyte

**Figure 3:**
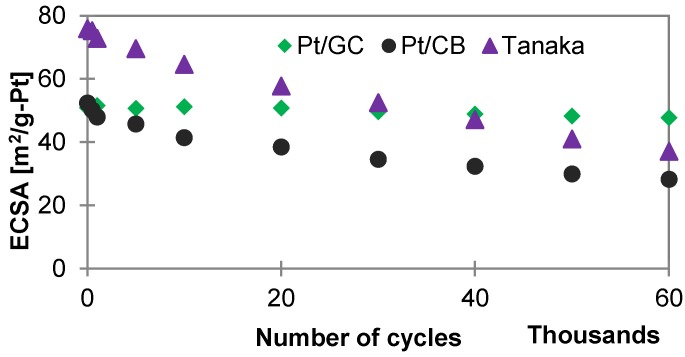
Comparison of support stabilities (in terms of ECSA survival rate) of the catalysts during potential cycle tests between 1.0 and 1.5 V vs. RHE in 0.5 M H_2_SO_4_ electrolyte at 500 mV/s voltage scan rate at 25 °C.

Figure 3 shows the degradation of the GC (Pt/GC)-, the CB (Pt/CB)-supported annealed catalyst, and the reference Tanaka catalyst in terms of loss of electrochemical surface areas (ECSA), with potential cycling between 1.0 V and 1.5 V vs. reversible hydrogen electrode (RHE) at 500 mV/s voltage scan rate in 0.5 M H_2_SO_4_ at 25 °C [36]. The initial ECSA of the Tanaka catalyst is found to be the highest, due to the lowest Pt particle size (<2 nm), however, after 60,000 potential cycles, the Pt/GC catalyst shows the highest (92%) electrochemical surface area survival rate (ECSA-SR), compared to the Pt/CB catalyst (54%) and the reference (49%) catalysts. Although the initial ECSA of both Pt/CB and Pt/GC catalysts are the same, there is a significant decrease of ECSA of the Pt/CB catalyst under cycling conditions which compromises its performance for long-term use, compared to the Pt/GC catalyst. Thus, a superior long-time uniform performance can be expected from the GC-supported catalyst.

#### 2.2.3. Pt Corrosion in Acid Electrolyte

**Figure 4:**
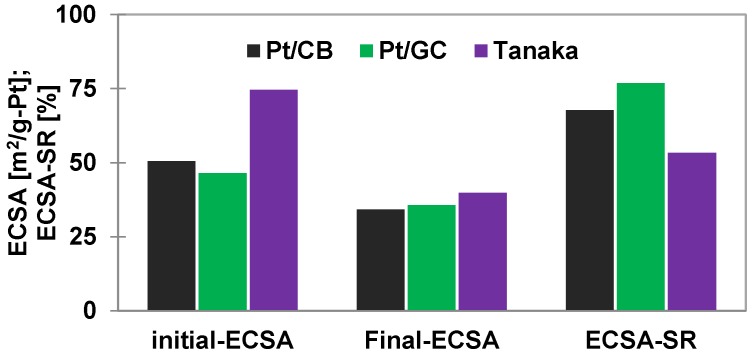
Comparison of Pt stabilities on the supports (in terms of ECSA survival rate) during potential cycling between 0.6 V and 1.0 V vs. RHE at 50 mV/s voltage scan rate in 0.5 M H_2_SO_4_ electrolyte at 25 °C.

Aim of this test is to investigate the catalyst (Pt) durability in 0.5 M H_2_SO_4_ under stressed conditions. Figure 4 displays the comparative Pt durability test results of the catalysts. The rates of Pt corrosion with potential cycling between 0.6 V and 1.0 V (Pt stressed areas) at 50 mV/s voltage scan rate are different for the supported catalysts. After 40,000 cycles, the ECSA-SR of the Pt/GC catalyst is the highest compared to the Pt/CB and the reference catalysts under the same cycling conditions. This could be due to the bigger particle sizes of Pt on the Pt/GC than the others [37]. 

#### 2.2.4. Oxygen Reduction Reaction (ORR) activities of the Catalysts

**Figure 5:**
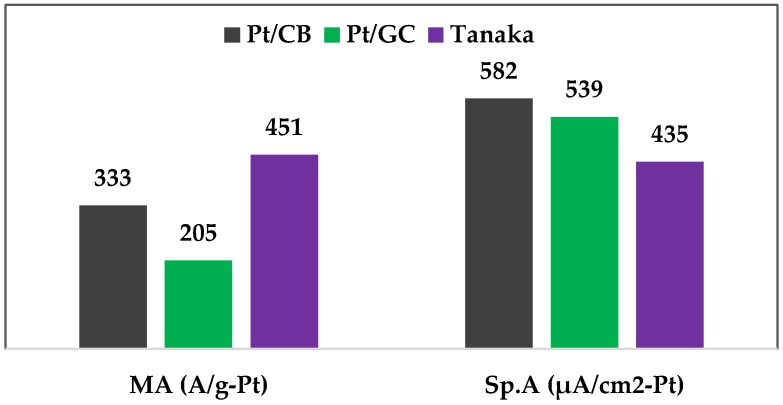
ORR activities of the supported catalysts at 0.9 V vs. RHE electrode in 0.1 M HClO_4_ electrolyte at 50 mV/s scan rate at room temperature.

The mass activities at 0.9 V vs. RHE electrode of the Pt/CB, Pt/GC, and the reference Tanaka catalysts are measured as 333, 205, and 451 A/g-Pt respectively (see Figure 5). Lower mass activies of the synthesized catalysts could be due to the bigger Pt particle sizes of the synthesized catalysts than the reference catalyst. However, higher specific activities of the synthesized catalyst are an indication of overall high ORR activities of these catalysts compared to the reference catalyst. 

#### 2.2.5. MEA Single Cell Performances

**Figure 6:**
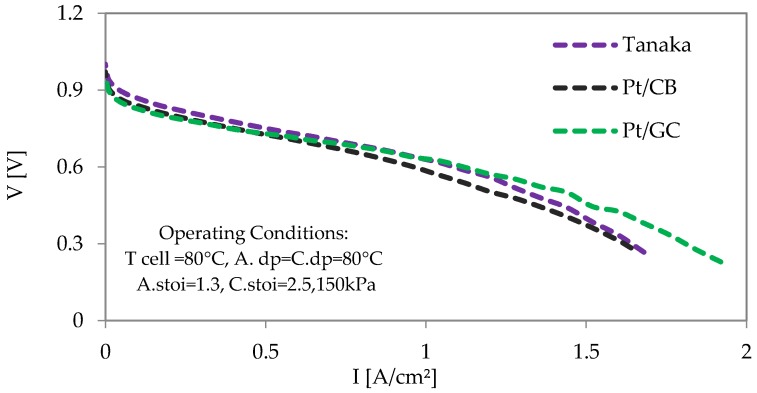
Comparative I–V characteristic curves of the MEAs using a Nafion^®^NR212 membrane with Pt/CB, Pt/GC, and Tanaka catalysts.

It has been shown in our previous work that each catalyst needed different amounts of ionomer on the catalyst layers in order to get the optimum performances [33]. It very much dependent on the BET surface areas of the support materials. Thus, optimization of the ionomer content on the catalyst layer for each catalyst was performed. Figure 6 shows the optimized results of MEA single cell tests in which optimum 37.5 wt % ionomer for Tanaka (carbon/ionomer = 0.74), 40 wt % ionomer for Pt/CB (carbon/ionomer = 0.82), and 29 wt % ionomer for Pt/GC (carbon/ionomer = 0.51) on the cathode catalysts layers have been used. All of the performance tests were carried out with using pure H_2_ as fuel, and air as oxidant. For up to 1 A/cm^2^ current densities, the Tanaka catalyst showed high voltages compared to the other catalysts, however, at high current densities, the Pt/GC catalyst showed higher voltages. For up to 0.5 A/cm^2^ current densities, MEAs with both Pt/CB and Pt/GC catalysts were operating at almost the same voltages, and eventually, larger ohmic drops and mass transport losses are observed from the Pt/CB catalyst than the Pt/GC catalyst. 

**Figure 7:**
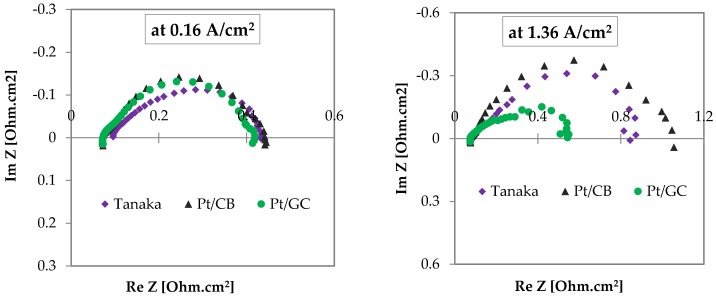
Nyquist plots at 0.16 and 1.36 A/cm^2^ current densities of the MEAs with Pt/CB, Pt/GC, and Tanaka catalysts under the same operation conditions as performance tests.

The Nyquist plots from impedance measurements uncover additional information of the MEAs diagonystics. Figure 7 shows that both at low (0.16 A/cm^2^) and at high (1.36 A/cm^2^) current densities, the MEA resistances of both CB- and GC-supported catalysts are the same. However, at high current density, the arc diameter, which is thought to be diffusion resistance, is significantly increased for the MEA prepared with the CB-supported catalyst, as well as the commercial Tanaka catalyst, which is probably the cause of low performances of these catalysts at high current densities, as we observed in the I–V characteristic curve. 

**Figure 8:**
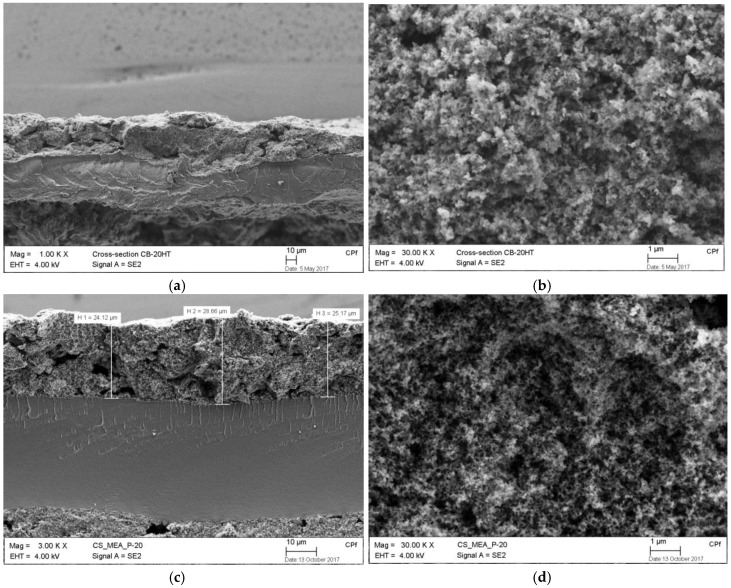

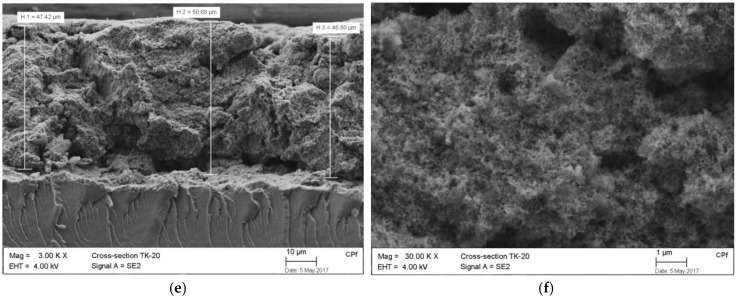
SEM images of the cross section of the MEAs, for Pt/CB (**a**,**b**), Pt/GC (**c**,**d**), and Tanaka (**e**,**f**) catalysts.

SEM images of the cross section of the cathodes also disclose the superiority of the MEA with Pt/GC catalyst in terms of electrode thickness and the morphologies (see Figure 8). Lower electrode thickness of the MEA with Pt/GC (26 µm) could be another reason for the improved mass transport phenomena, compared to the MEA with Pt/CB (34 µm) and the Tanaka (48 µm) catalysts. 

## 3. Discussion

In this work, the comparative performance stabilities of supported Pt electrocatalysts on a typical CB and GC support were investigated. Pt nanoparticles of almost the same particle sizes were successfully deposited on the GC and the CB supports using the already developed modified polyol process. The total Pt loadings of the supported catalysts were also within good ranges, while the target was 20 wt % Pt-supported catalysts. 

After 60,000 potential cycles between 1.0 V and 1.5 V vs. RHE in 0.5 M H_2_SO_4_ at 500 mV/s scan rate at 25 °C, the ECSA-SR of the Pt/GC catalyst was higher than both of Pt/CB and the reference Tanaka catalysts. 

Pt stability is mainly dependent on the particle sizes of Pt. However, metal support interactions have some influence on Pt stability, as we found in our previous work [33]. In this work, Pt stability on GC in 0.5 M H_2_SO_4_ was found to be slightly higher than on the traditional CB support after 40,000 potential cycles between 0.6 V and 1.0 V vs. RHE at 50 mV/s at 25 °C, probably due to the effect of Pt particle sizes.

ORR activity of Pt/GC is found to be lower than both of the Pt/CB and the Tanaka catalysts. Nevertheless, MEA single cell performance of the former is highly comparable with commercial Tanaka catalyst. Due to low electrode thickness and high porosity, mass transport resistance of the GC-supported catalyst was improved. Thus, it showed promising performance among all other catalysts which were investigated. 

In this work, stable cathode catalyst supports were investigated. The use of the same support as anode will be the next part of our continuous development plan. 

## 4. Materials and Methods 

In this work, a CB support (Vulcan XC72, Cabot Corporation, Billerica, MA, USA) and a GC support (Timcal-167, Imerys Graphite & Carbon, Bodio, Switzerland) were chosen for investigation. As received GC was hydrophobic with low BET surface area. In order to increase its surface properties, oxidation at 600 °C for 90 min in an air oven was performed. CB material was used without any further treatment. The BET surface areas of the materials were measured using a Sorptomatic 1990 instrument (Thermo Scientific Inc, Waltham, MA, USA), while N_2_ was used as adsorbent. The electronic conductivities of the materials were measured via our homemade four-point measuring device, by passing through AC current and measuring the voltage in a known thickness of the materials. The supported Pt electrocatalysts of targeted 20 wt % Pt loading were prepared by using an already developed modified polyol process [33]. An X-ray diffraction technique with a Siemens D5000 (Bruker AXS, Karlsruhe, Germany) and TOPAS software (version 5) were used to determine the Pt particle size of the synthesized catalysts. The contents of Pt on the supported catalysts were measured via ICP-OES analysis (Acros FHS12, Spectro Analytical Instruments GmbH, Kleve Germany). 

Degradation test of the supports and the Pt were performed in 0.5 M H_2_SO_4_ at 25 °C in a three-electrode configuration, using a Zahner IM6 potentiostat (Zahner-elektrik GmbH & Co. KG, Kronach, Germany) while using Hg/HgSO_4_ as reference electrode, Pt wire as counter electrode, and 10 µg Pt-loaded working electrode (WE). The WE was prepared by placing the required amount of ink on a glassy carbon disk and drying it in air. Ink for the WE was prepared by taking 10 mg catalyst powder and 5 mL solvent (0.02 wt % Nafion^®^ in water) in a glass vial with a magnetic stirrer, which was then stirred for 2 min, followed by sonication for 15 min. Initially, the electrode surface was cleaned via potential cycling between 0.05 V and 1.2 V vs. RHE at 200 mV/s scan rate until getting a reproducible CV, followed by taking CV at 50 mV/s within the same ranges to measure initial electrochemical surface areas (ECSA). The support corrosion test was performed by potential cycling between 1.0 V and 1.5 V vs. RHE at 500 mV/s voltage scan rate [36], while the Pt corrosion test was performed by potential cycling between 0.6 V and 1.0 V vs. RHE at 50 mV/s voltage scan rate [33]. 

A rotating disk electrode (RDE) setup with a biologic potentiostat (SP-150) and an RDE 710 rotator (Gamry instruments, Warminster, PA, USA) were used to measure the ORR activities of the catalysts. The measurement was performed in 0.1 M HClO_4_ at 50 mV/s voltage scan rate at room temperature while using Hg/HgSO_4_ as RE, Pt wire as CE, and 1.4 µg Pt-loaded gold electrode as WE. The measurement procedure was the same as reference [33]. 

MEAs were prepared via catalyst-coating membrane techniques in a Nafion^®^NR212 (50.8 µm) membrane [38]. Cathodes of the MEAs were prepared with the supported catalysts to be investigated while keeping the anodes unchanged with a commercial Tanaka catalyst (19.3 wt % Pt on CB) for all MEAs. The Pt loading on both electrodes were 0.3 mg/cm^2^. A standard FC technology single cell with triple serpentine flow fields and 25 cm^2^ active surface areas was used to conduct MEA performance tests. The MEA performances and EIS measurements were carried out using an already developed testing procedure [38]. After the tests, the MEAs were cut using N_2_ freezing in order to investigate the cross-sectional morphology of the electrodes via scanning electron microscopy (Zeiss SEM/FIB NVision 40, Zeiss, Oberkochen, Germany).
